# Interplay Between Systemic Metabolic Cues and Autonomic Output: Connecting Cardiometabolic Function and Parasympathetic Circuits

**DOI:** 10.3389/fphys.2021.624595

**Published:** 2021-03-11

**Authors:** Liliana Espinoza, Stephanie Fedorchak, Carie R. Boychuk

**Affiliations:** Department of Cellular and Integrative Physiology, Long School of Medicine, University of Texas Health San Antonio, San Antonio, TX, United States

**Keywords:** autonomic, metabolic, parasympathetic, cardiovascular disease, brainstem, vagus

## Abstract

There is consensus that the heart is innervated by both the parasympathetic and sympathetic nervous system. However, the role of the parasympathetic nervous system in controlling cardiac function has received significantly less attention than the sympathetic nervous system. New neuromodulatory strategies have renewed interest in the potential of parasympathetic (or vagal) motor output to treat cardiovascular disease and poor cardiac function. This renewed interest emphasizes a critical need to better understand how vagal motor output is generated and regulated. With clear clinical links between cardiovascular and metabolic diseases, addressing this gap in knowledge is undeniably critical to our understanding of the interaction between metabolic cues and vagal motor output, notwithstanding the classical role of the parasympathetic nervous system in regulating gastrointestinal function and energy homeostasis. For this reason, this review focuses on the central, vagal circuits involved in sensing metabolic state(s) and enacting vagal motor output to influence cardiac function. It will review our current understanding of brainstem vagal circuits and their unique position to integrate metabolic signaling into cardiac activity. This will include an overview of not only how metabolic cues alter vagal brainstem circuits, but also how vagal motor output might influence overall systemic concentrations of metabolic cues known to act on the cardiac tissue. Overall, this review proposes that the vagal brainstem circuits provide an integrative network capable of regulating and responding to metabolic cues to control cardiac function.

## Introduction

While sympathoexcitation may be widely accepted as a hallmark of the pathogenesis of cardiovascular disease, decreased parasympathetic, or vagal, tone is linked to a broad spectrum of diseases, including cardiac arrhythmias, coronary heart disease, and heart failure, and is an accurate predictor of morbidity and mortality in humans and animals (Barkai and Madacsy, 1995; La Rovere et al., 1998; Nolan et al., 1998; Thayer and Lane, 2007; Franciosi et al., 2017). Experiments conducted more than 150 years ago first established the anti-arrhythmogenic effect of vagal stimulation (Lown and Verrier, 1976). With later seminal work in dogs demonstrating that blocking muscarinic acetylcholine receptors abolished this effect of vagal nerve stimulation, there is now convincing evidence that increased vagal nerve activity can ameliorate poor cardiac function (Schwartz et al., 1984; Vanoli et al., 1991).

Significant clinical evidence also implicates metabolic diseases (i.e., obesity and diabetes) as independent risk factors for the development of cardiovascular disease (Benjamin et al., 1994; Bonow and Eckel, 2003; Baek et al., 2017; Aune et al., 2018; Rawshani et al.,2018a,b). Notably, diseases of metabolism also associate with autonomic dysfunction (Barkai and Madacsy, 1995; Vinik et al., 2013). While the mechanism(s) linking metabolic disorders with cardiac dysfunction remain heavily debated, the role of the central autonomic nervous system in the orchestration of cardiometabolic homeostasis warrants a discussion on its potential role as a neuromechanistic link between metabolic signaling and cardiovascular function. While the heart as an isolated entity is important, it is critical to understand that it represents a single part of a larger system.

Although a large scale clinical investigation suggested that chronic vagal stimulation did not improve cardiac function in patients with heart failure (Zannad et al., 2015), this study emphasizes the need for better strategies to target efferent cardiac vagal output given the known disadvantages of activating non-cardiac vagal motor neurons and vagal sensory afferent fibers (Buckley et al., 2015). Therefore, understanding the neuronal modulation of cardiac activity could provide novel mechanistic details into the pathogenesis and treatment of cardiovascular disease. This idea is further reinforced since advanced neural modulation techniques have proven effective treatments for other cardiovascular diseases, including cardiac arrhythmias (Kapa et al., 2010; Shen et al., 2011).

This review then will focus on autonomic function in the context of cardiometabolic physiology, particularly as it relates to vagal motor output. Therefore, it will aim to define basic brainstem circuits and the influence of metabolic signaling on plasticity within these circuits. It will hopefully compel more investigations into how vagal motor output is both affected by and an effector of metabolic cues.

## Parasympathetic Circuits and Their Regulation of Heart Rate

Ever since the first description of cardiac innervations in the 19th century (Hirsch, 1970), significant work has been done to map cardiac autonomic networks. Therefore, it is well established that the heart is innervated by two distinct branches of the autonomic nervous system, the sympathetic and parasympathetic. Despite the well acknowledged understanding of these two divisions, the paradigm generally used in textbooks and cardiology reviews overly simplifies the regulation of cardiac function as dependent almost exclusively on sympathetic activity. Therefore, our current dogma discounts the contributions of the parasympathetic nervous system to cardiac function. All of this despite consensus that in most vertebrates, including humans, the activity of myogenic pacemaker sinoatrial (SA) nodal cells is largely regulated by the tonic, inhibitory influence of parasympathetic motor output, making vagal tonus the predominant determinant of resting heart rate (Gordan et al., 2015).

**FIGURE 1:**
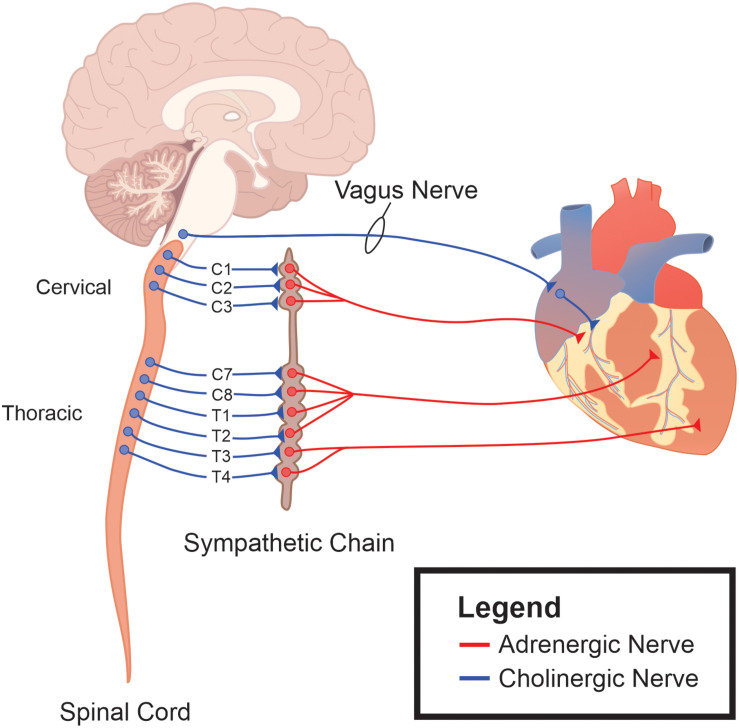
The autonomic nervous system as an integrative control center for cardiac control. Sympathetic ganglia are located in the intermediolateral (IML) cell column of the thoracic spinal cord. The sympathetic ganglia send prominent projections to both cardiac tissue and the vascular system. Efferent parasympathetic, or vagal, originate within the brainstem and project to the epicardial fat sac in close apposition to cardiac tissue. It is these ganglia, along with their postganglionic fibers and their interconnections, that represent the final pathway for autonomic regulation of cardiac function.

Vagal motor innervation of cardiac tissue is comprised of cholinergic, preganglionic motor neurons whose cell bodies are located in the brainstem (Figure 1). These preganglionic neurons send their axons through the vagus nerve, and synapse onto intracardiac postganglionic motor neurons. Intracardiac vagal postganglionic neurons are also cholinergic, and traditionally thought to be subservient relay stations since the majority of these neurons lose their ability to generate spontaneous electrochemical activity when preganglionic motor neuron innervations are severed (Armour, 2008). Consequently, cardiac-related vagal efferent nerve activity is initiated at the soma of preganglionic cardiac vagal motor neurons, and alterations in their firing properties affect vagal nerve motor efferent output.

These preganglionic cardiac vagal motor neurons originate from two brainstem regions: the nucleus ambiguus (NA) and the dorsal motor nucleus of the vagus (DMV) (Standish et al., 1994; Massari et al., 1995; Cheng and Powley, 2000). The vast majority of cardiac innervation (approximately 80%) in higher mammals originates from motor neurons located in the NA. These NA cardiac vagal motor neurons uniformly exert a strong cardioinhibitory influence on heart rate (McAllen and Spyer, 1977; Geis and Wurster, 1980; Gilbey et al., 1984). Importantly, NA neurons are intrinsically silent, implicating synaptic input as a strong regulator of their overall activity (Mendelowitz, 1996). Cardiac vagal motor neurons in the NA are also critical to cardiovascular disease development. Studies conducted in animal models of obstructive sleep apnea, for example, demonstrated that diminished vagal output activity and blunted baroreflex control of heart rate are due to changes in cardiac vagal motor neurons located in the NA, and not due to changes in intracardiac ganglia activity or innervation (Gu et al., 2007; Lin et al., 2007; Yan et al., 2009).

Little is known, however, about the innervation arising from the cardiac-projecting neurons residing within the DMV. Retrograde tracing studies indicate that cardiac motor neurons do originate in the DMV (Calaresu and Cottle, 1965; Sturrock, 1990; Standish et al., 1994), providing instrumental evidence of the existence of DMV cardiac-projecting motor neurons. However, while several studies reported cardioinhibitory activity from the DMV (Schwaber and Schneiderman, 1975; McAllen and Spyer, 1977), others suggest a lack of an effect on heart rate (Geis and Wurster, 1980). It is important to note that the majority of previous studies relied heavily on techniques with limited spatial precision and specificity. This is critical to our understanding of the DMV’s contribution to heart rate since non-motor inhibitory interneurons exist within this nucleus as well (Jarvinen and Powley, 1999; Gao et al., 2009). Using techniques with improved specificity, like optogenetics, activation of DMV motor neurons increased cardiac ventricular contractility and enhanced exercise endurance in rodents (Machhada et al., 2017), protected ventricular cardiomyocytes from ischemic/reperfusion injury (Mastitskaya et al., 2012), and altered the electrical properties of cardiac tissue (Machhada et al., 2015). Importantly, these latter two results were independent of changes in heart rate, providing key experimental evidence that the DMV might be the source of vagal nerve-dependent coronary artery dilation (Reid et al., 1985; Kovach et al., 1995). However, these studies utilized a viral expression system for Phox2 cholinergic DMV neurons and could not distinguish between cardiac-projecting DMV neurons and those that project to other visceral organs. As discussed later in this review, DMV vagal motor neurons are critical in the regulation of metabolic cues, such as insulin and glucagon, and there is a possibility that these secondary humoral factors played a role. However, both anatomical and more traditional stimulation approaches do support a role for DMV activity in direct regulation of ventricular cardiomyocyte regulation (Dickerson et al., 1998; Lewis et al., 2001; Ulphani et al., 2010).

We also know relatively little in terms of the electrophysiological properties and upstream signaling network governing the activity of cardiac-projecting DMV neurons. While cardiac NA neurons have low resting membrane potentials and are silent when devoid of synaptic input, DMV neurons with other visceral organ targets exhibit a slow pace-making current (Browning et al., 1999). The presence of pace-making currents fundamentally alters the relationship of neuronal excitability and synaptic input. Therefore, if cardiac-projecting DMV neurons possess similar pace-making currents, these neurons could serve a unique role in cardiovascular autonomic regulation. In support of such a role, synaptic input to cardiac-projecting DMV neurons after heart failure undergoes unique signaling plasticity compared to cardiac-projecting NA neurons (Cauley et al., 2015). Still, given the existing controversy over the contribution of cardiac DMV motor neurons to cardiac regulation, future studies will continue to provide a more accurate depiction of cardiac parasympathetic innervation and regulation.

### Central Brainstem Parasympathetic Circuits

**FIGURE 2:**
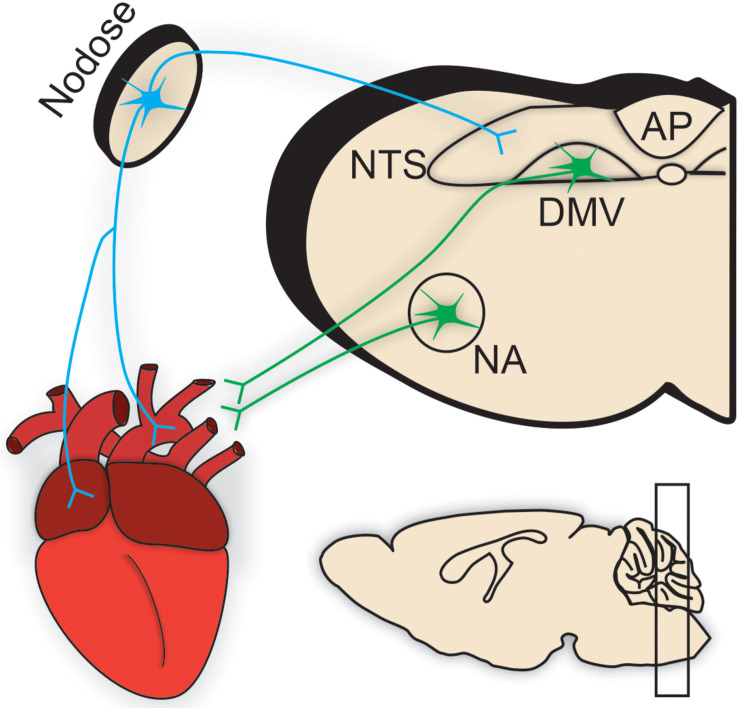
Brainstem parasympathetic circuits. Efferent parasympathetic, or vagal, innervation (illustrated in green) to cardiac tissue originates from preganglionic brainstem motor neurons predominantly residing within the nucleus ambiguus (NA) and the dorsal motor nucleus of the vagus (DMV). These preganglionic neurons send their projections through the vagus nerve to synapse onto intracardiac parasympathetic ganglia located in close apposition to cardiac tissue [e.g., pacemaker nodal cells in the sinoatrial (SA) node].Conversely, sensory afferent information is carried by sensory neurons (shown in blue) located throughout the heart, especially in ventricular and atrial tissues, and aortic arch. Most prominent in cardiometabolic regulation are those vagal afferents in the intrathoracic nodose ganglia. Nodose ganglia afferent inputs synapse directly onto the nucleus tractus solitarius (NTS). NTS neurons [and the circumventricular organ, area postrema (AP)] integrate this sensory information from the heart (in addition to other peripheral sensory information from viscera important in the regulation of respiratory, gastrointestinal, and metabolic homeostasis). This integrated sensory information is either relayed to descending vagal motor neurons in a pathway termed “vago-vagal” reflexes or to upstream brain regions for further processing and integration. Therefore, the brainstem is a critical location for the orchestration of central motor control of cardiovascular function.

Regardless of the location and electrophysiological properties of cardiac vagal motor neurons, upstream central brainstem signaling is critical to their final motor output. Centrally-mediated autonomic motor control of the cardiovascular system is sensitive to various sensory afferent information carried in large part by peripheral neurons located in the intrathoracic nodose ganglia, which synapse in the nucleus tractus solitarius (NTS) (Figure 2; Armour, 2008). First and second order NTS neurons integrate the excitatory, glutamatergic information from these peripheral afferents to influence the activity of downstream parasympathetic motor neurons. Traditionally, the caudal aspect of the NTS contains the majority of cardiovascular-related afferent synapses, following a general topographic organization (Loewy and Spyer, 1990). Important to cardiovascular regulation is the baroreceptor reflex. Increases in arterial blood pressure result in the activation of baroreceptors, which then convey this information to NTS neurons to initiate a reflexive decrease in heart rate. This is primarily achieved through increases in cardiac vagal nerve activity, and requires little to no inhibition of cardiac sympathetic nerve activity (Dun et al., 2004). Vagal motor output is also critical to other cardiovascular reflex responses including respiratory sinus arrhythmia (Dergacheva et al., 2010).

However, there is considerable overlap in visceral organ-based topography within the NTS with sensory afferent innervations from other organ systems (i.e., pulmonary stretch receptors (Katona and Jih, 1975; Taylor et al., 2014) and arterial chemoreceptors (Accorsi-Mendonca and Machado, 2013), including subdiaphragmatic organs involved in metabolic regulation (Browning et al., 1999; Browning and Travagli, 2011; Taylor et al., 2014). The NTS and its neighboring circumventricular organ, area postrema (AP), are also implicated as central metabolic sensors, including neurons that respond to insulin (Ruggeri et al., 2001; Blake and Smith, 2012), glucose (Balfour et al., 2006; Lamy et al., 2014; Boychuk et al., 2015a; Roberts et al., 2017), ghrelin (Cui et al., 2011), and leptin (Barrera et al., 2011). Importantly, the ability of these brain regions to directly sense metabolic state can influence peripheral physiology (Ritter et al., 2000; Ferreira et al., 2001; Lamy et al., 2014; Boychuk et al., 2019). For example, in terms of cardiometabolic behavior, insulin microinjections into the NTS decreased the activity of baroreceptor-sensitive NTS neurons (McKernan and Calaresu, 1996; Ruggeri et al., 2001), and despite only limited effects on resting heart rate (McKernan and Calaresu, 1996; Krowicki et al., 1998), insulin in the NTS significantly reduced the baroreflex response (McKernan and Calaresu, 1996; Ruggeri et al., 2001). Similar interactions likely occur with other metabolic signals since leptin has also been implicated in a reduction of baroreflex responses (Arnold and Diz, 2014). Therefore, information related to metabolic status is quickly and efficiently integrated into cardiovascular regulatory networks within the dorsal hindbrain. These integrated circuits are likely evolutionary mechanisms developed to allow for gross matching of cardiac output to both metabolism and respiration (Castro et al., 2003; Srivastava, 2012; Taylor et al., 2014). Therefore, the brainstem represents an integrative network of neurons responsible for sensing systemic cardiorespiratory and metabolic states and coordinating motor neuron control of such systems, resulting ultimately in the maintenance of internal homeostasis.

## The Influence of Metabolic Disruptions on Parasympathetic Function

Metabolic dysregulation (i.e., obesity and diabetes) is an independent risk factor for the development of cardiovascular disease (Benjamin et al., 1994; Bonow and Eckel, 2003; Baek et al., 2017; Aune et al., 2018; Rawshani et al.,2018a,b). While metabolic disorders come with complex multisystem morbidities, longitudinal studies conducted in human patients have long indicated that vagal dysfunction is first in the autonomic dysfunction sequelae and occurs prior to overt cardiac complications (Ewing et al., 1980; Vinik et al., 2011) or induction of fasting hyperglycemia (Wu et al., 2007). Despite consensus that autonomic dysfunction is a hallmark of most cardiovascular diseases, the precise role of autonomic cardiac-related activity in the context of metabolic disruption remains debated. This section will discuss the role of two important metabolic cues, diet and inflammation, and their potential to alter vagal regulatory circuits.

### High Fat Diet as a Metabolic Disruption

The nuances surrounding different diets and their relationship to cardiovascular disease are best reviewed elsewhere (Sacks et al., 2017). However, it has long been recommended that individuals wishing to reduce their risk of cardiovascular disease should reduce dietary saturated fatty acid consumption. This evidence includes randomized clinical trials reporting improvements in the incidence of poor cardiac outcomes, including sudden death, after reductions in saturated fat intake (Dayton et al., 1962; Leren, 1970). Importantly, animal models exposed to high dietary fat content mimic several characteristics of cardiovascular disease (Dobrian et al., 2000), and while the mechanisms mediating the effects of high fat diet are still up for debate, it remains possible that the effects of high fat diets on vagal circuits contribute to the development of cardiovascular disease.

In animal models, diets high in saturated fats can induce tachycardia (Van Vliet et al., 1995; Dobrian et al., 2001; Bruder-Nascimento et al., 2017), even if only a mild tachycardia (Carroll et al., 2006). Moreover, in studies that failed to identify this tachycardia, there was still significant evidence for reduced cardiac vagal tone (Williams et al., 2003; Chaar et al., 2016). This reduction in vagal drive includes an abolishment of vagal responsivity during the baroreflex (Van Vliet et al., 1995). Historically, decreased cardiac vagal tone during disease (purely cardiovascular or metabolic in nature) is attributed to vagal neuropathy (Bolinder et al., 2002; Horowitz et al., 2002). However, emerging evidence from other peripheral nerve systems demonstrates that lack of neuronal activity itself can eventually lead to neuronal degeneration and neuropathy (Gibson et al., 2014). Similar experimental evidence has not been examined for vagal motor output, but at the very least, the reduced baroreflex activity appears consistent with a reorganization of central cardiovascular circuits that results in a lack of vagal motor output early in disease progression (McCully et al., 2012).

Unfortunately, the mechanism(s) responsible for vagal circuit reorganization of the brain regions involved are still largely unknown. There are now substantial data implicating vagal afferents and the NTS as important sites in the effects of high fat diet in the context of feeding regulation (de Lartigue et al., 2011), suggesting it as a possible location for the effects of high fat diet on cardiovascular regulation. To our knowledge, only a limited number of studies examine the role of high fat diet in the regulation of cardiac-projecting vagal motor neuron excitability and function. However, these studies do support the idea that reduced vagal drive originates from reduced vagal motor activity. High fat diet decreases c-Fos expression in the NA, suggesting a reduction in neuronal activation compared to normal chow controls (Alsuhaymi et al., 2017). Although to date there is no data on the effects of high fat diet on cardiac-projecting DMV neurons, high fat diet for 12 weeks reduces gastric-projecting DMV motor neuron excitability as measured by whole-cell patch-clamp techniques (Browning et al., 2013). Similarly, perinatal exposure to high fat diet increases GABAergic inhibitory synaptic signaling in DMV neurons (McMenamin et al., 2018; Clyburn et al., 2019). Interestingly, other conditions of metabolic dysregulation of vagal motor neurons suggest that altered synaptic signaling occurs through inappropriately low trafficking of select synaptic receptor populations out of the cellular membrane (Zsombok et al., 2011; Boychuk et al., 2015b), but this mechanism has yet to be confirmed for high fat diet or cardiac-projecting vagal motor neurons. Taken together, it remains possible that decreased neuronal activity within vagal motor neurons themselves eventually leads to reductions in vagal motor efferent drive.

The case for early inhibition of the vagal motor system can only be made through longitudinal evaluations of consumption of high fat diets (that includes earlier timepoints in the feeding paradigms). While a significant amount of research is appropriately dedicated to investigating the role of long-term high fat diet and ultimately the obesity that follows long term consumption of these diets, there is increasing evidence that consumption of high fat for a few days can affect neuronal function. Part of this reevaluation includes the emerging concept that metabolic challenges lead to neural adaptions, rendering the brain insensitive to future metabolic cues (Beutler et al., 2020; Mazzone et al., 2020). Importantly, some aspects of adaptive neural plasticity occur quickly, for example after a single bout of exercise (He et al., 2018). There are several investigations suggesting similar effects likely exist in vagal circuits. For example, patients with type 2 diabetes first show impaired glucose intolerance during the vagally-mediated component of insulin release (Gallwitz et al., 2013), and reducing dietary fat intake increases cardiac vagal activity even in non-obese patients (Pellizzer et al., 1999). In animal studies, DMV vagal motor neurons exhibit increased glutamatergic neurotransmission after short term (less than 5 days) high fat consumption (Clyburn et al., 2018). Although it has not been determined if this increase in glutamatergic signaling can drive disease pathogenesis or is compensatory in nature (for say elevated inhibitory signaling), it does confirm that high fat diet alters synaptic signaling to vagal efferent motor neurons earlier than previously considered. Reduced parasympathetic motor activity during early disease progression would not be unique to metabolic dysfunction. Reductions in parasympathetic tone occurred early and often preceded increased sympathetic activity in both animal models of and patients with heart failure (Ishise et al., 1998; Motte et al., 2005). Therefore, these types of investigations into the early influence of diets may help elucidate the neuromechanism(s) through which high fat diet contributes to the pathogenesis of cardiovascular disease (Fleming, 2002; Hartnett et al., 2015).

### Inflammation as a Metabolic Disruption

Chronic inflammation is also considered a significant risk factor for the development of cardiovascular disease (de Kloet et al., 2013). Of particular importance is the role of neuroinflammation through brainstem autonomic mechanisms. As the brain’s resident immune cells (Ransohoff and Brown, 2012), microglia play an important role in this signaling within the brainstem. For example, microglial activation is noted within the NTS in both experimentally-induced type-1 diabetes (Rana et al., 2014) and high fat diet animal models (Minaya et al., 2020). Additionally, vagal afferents are directly activated by proinflammatory signals (Besedovsky et al., 1986; Waise et al., 2015), suggesting that in addition to direct effects on neurons residing within the brainstem, inflammation can modulate autonomic feedback control mechanisms by modulation of vagal afferent signaling.

Another emerging modulator of central cardiovascular regulatory networks is the most abundant glial cell in the central nervous system, the astrocyte (Martinez and Kline, 2021). Originally identified for their importance in the generation of the blood brain barrier, astrocytes not only contain multiple immune receptors, but respond to a number of immune signals (Colombo and Farina, 2016). Astrocytic activity in the brainstem can influence cardiovascular function (Martinez et al., 2020). Importantly, activation of astrocytes is linked to cardiovascular dysregulation after high fat diet consumption (Worker et al., 2020), and astrocyte signaling is required for high fat diet-induced hyperphagia and obesity (Douglass et al., 2017).

Indirect pathways of neuroinflammatory activation are also implicated in cardiovascular (dys)function. The renin-angiotensin system (RAS) is classically considered an endocrine regulator of the cardiovascular system. However, it is now recognized that components of the RAS system are present within the brain, including the brainstem (Cuadra et al., 2010), and RAS signaling is elevated during metabolic disorders (Giacchetti et al., 2005). Convincing evidence now exists suggesting that central RAS signaling is critical to the development of hypertension through sympathetic activation of peripheral inflammation (de Kloet et al., 2013). However, convincing evidence now implicates the activation of vagal signaling in reducing the inflammatory response (Pavlov and Tracey, 2012), and the DMV was recently identified as the critical brain region for the generation of this response (Kressel et al., 2020). Moreover, elevated RAS signaling also increases the permeability of the blood-brain barrier (Biancardi et al., 2014). Therefore, brainstem autonomic circuits are likely exposed to other systemically circulating factors (e.g., inflammatory cytokines). Therefore, it remains possible that reduced vagal signaling promotes the inflammation typically seen during chronic metabolic conditions, like diabetes and obesity.

## The Influence of Parasympathetic Signaling on Metabolic Cues

To fully understand the role of perturbations in metabolic cues on cardiac function, it is important to understand the role of autonomics in mediating the concentrations and sensitivities of metabolic hormones with known cardiac modulatory abilities. It is worth mentioning that much of our understanding of the role of metabolic signaling perturbations has been insulin-centric. However, it is becoming evident that metabolic diseases come with a complex hormonal milieu. As such, there have been calls for a more diverse focus, such as on glucagon (Unger and Cherrington, 2012; Lee et al., 2014). Therefore, this section will focus on the role of the parasympathetic nervous system to regulate the concentration of and sensitivity to key metabolic cues which have established roles in cardiac regulation, namely insulin, glucagon, and GLP-1.

### Insulin

First discovered more than 100 years ago, insulin is secreted from pancreatic beta cells in response to high blood glucose concentrations and its role in metabolic dysregulation is well established. Insulin receptors are widely expressed throughout the cardiovascular system, including cardiac tissue (Wang et al., 1997; Muniyappa et al., 2007; Riehle et al., 2014). Insulin resistance in cardiac tissue is suggested as a major risk factor for cardiovascular disease (Paternostro et al., 1996), and insulin resistance appears in cardiovascular diseases not explicitly related to energy homeostatic changes in metabolism, like heart failure (Mori et al., 2012; Zhang et al., 2013). Elevated sympathetic nervous system activity has been linked to the development of insulin resistance in cardiac tissue (Morisco et al., 2005; Mangmool et al., 2016), and to our knowledge similar studies have not been conducted for vagal signaling in cardiac tissue. This is a significant gap in knowledge since vagal signaling promotes insulin sensitivity in other peripheral organs, and the loss of vagal activity associates with insulin resistance (Xie et al., 1993; Xie and Lautt, 1994, 1995, 1996), leading some to conclude that vagal activity is critical to overall insulin sensitivity (Lautt, 1999).

The parasympathetic nervous system also plays an important role in the secretion of insulin. Preganglionic vagal innervation to the pancreas originates from the DMV, with little known contribution from the NA (Berthoud and Powley, 1990; Love et al., 2007; Rodriguez-Diaz et al., 2011; Chandra and Liddle, 2013). These DMV preganglionic fibers terminate on cholinergic intrapancreatic ganglia that influence pancreatic beta cells to release insulin (Thorens, 2014). However, intrapancreatic vagal ganglia also signal through non-adrenergic non-cholinergic (NANC) pathways such as nitric oxide or vasoactive intestinal polypeptide signaling (Wang et al., 1999; Love et al., 2007; Di Cairano et al., 2016) and these pathways can trigger insulin release as well (Mussa et al., 2011). While vagal motor innervation to the pancreas is relatively limited (Berthoud and Powley, 1990), there is evidence that abnormal, vagally-mediated insulin release is an early marker of diabetes (Gallwitz et al., 2013), suggesting that this may ultimately contribute to the pathogenesis of cardiovascular disease with strong metabolic connections.

### Glucagon

Serving in opposition to insulin (Cherrington and Vranic, 1971), glucagon is secreted from pancreatic alpha cells in response to low blood glucose concentrations and works to increase glucose production in and secretion from the liver. In humans, a single bolus of glucagon increases heart rate (Parmley et al., 1968), which has made it a useful therapy for reversing the effects of many cardioinhibitory drugs (White, 1999). However, hyperglucagonemia is present in metabolic disorders, including type 1 and 2 diabetes (Muller et al., 1973; Ichikawa et al., 2019) and has received renewed attention in terms of its role in the hyperglycemia associated with these conditions. Therefore, the influence of vagal activity on glucagon secretion may also influence cardiac regulation.

Unlike insulin, however, our understanding of how the parasympathetic nervous system regulates glucagon secretion is more controversial. Despite evidence of parasympathetic innervation to the pancreas, very few studies have dissected out the precise innervation to alpha cells themselves (Rodriguez-Diaz et al., 2011). Using more functional approaches, there is evidence that similar to insulin, vagal stimulation increases glucagon release (Ionescu et al., 1983; Ahren and Taborsky, 1986; Berthoud et al., 1990). This has been proposed as the mechanism behind the cephalic response to food consumption when glucagon is released to prevent an insulin-induced drop in blood glucose before food is ingested (Berthoud and Powley, 1990). More recent experiments continue to link increased glucagon concentration with increased vagal nerve bundle activity through brainstem glucose sensing mechanisms (Lamy et al., 2014). However, attempts to dissect out efferent vs. afferent vagal stimulation have suggested that increases in blood glucose levels (as would occur when glucagon is released) are achieved by efferent inhibition not activation (Meyers et al., 2016). Moreover, reduced preparations such as whole-cell patch-clamp combined with *in vivo* glucagon measures also suggest that inhibition of DMV motor activity increases glucagon concentrations (Boychuk et al., 2019). Taken together, these latter data provide evidence that parasympathetic efferent tone might serve as a brake for glucagon secretion. Therefore, the renewed interest in investigations into the role of glucagon in glucose homeostasis must continue to dissect out the autonomic nervous system’s contribution to its regulation since these types of studies likely have importance far beyond just glucose homeostasis.

### Glucagon-Like Peptide

The small peptide hormone glucagon-like peptide-1 (GLP-1) is an incretin hormone produced from L-cells within the intestine and released during digestion of fat and carbohydrates (Drucker, 2001). Upon release, GLP-1 acts to increase gastric volume, inhibit gut motility, and increase insulin secretion (Lim and Brubaker, 2006). GLP-1 receptors are also present in cardiac tissue (Pyke et al., 2014; Baggio et al., 2018) and GLP-1 signaling in cardiac tissue results in increased heart rate *in vivo* (Hayes et al., 2008; Baggio et al., 2017) and *in vitro* (Zhao, 2013; Ang et al., 2018).

However, the action of GLP-1 in cardiac regulation may be more complex (Ussher and Drucker, 2012). There is consensus that vagal signaling positively influences the release of GLP-1 (Anini and Brubaker, 2003) since either pharmacological inhibition of vagal signaling or vagotomy reduces serum concentration of GLP-1 (Rocca and Brubaker, 1999; Anini and Brubaker, 2003). However, acute bolus of GLP-1 induces a tachycardia through activation of GLP-1 receptors in central sympathetic regulatory networks, and not through activity at cardiac tissue itself (Hayes et al., 2008; Ghosal et al., 2013; Baggio et al., 2017). Moreover, GLP-1 receptor agonist injected into the NA not only decreased indices of cardiac vagal activity, but also depressed neurotransmission to cardiac vagal motor neurons (Griffioen et al., 2011), suggesting that NA neurons could also mediate the GLP-1-induced tachycardia. Therefore, while vagal activation likely supports the release of GLP-1, GLP-1 may negatively feedback on cardiac regulation to decrease vagal drive and induce sympathoexcitation. Adding further complexity, despite inducing a tachycardia and sympathoexcitation, GLP-1 mimetics not only improve glucose intolerance but provide cardioprotective benefits (Barnett, 2012; Zhao, 2013; Del Olmo-Garcia and Merino-Torres, 2018). These paradoxical effects of GLP-1 signaling could be the result of species-specific biology, as well as differences in approaches and outcomes tested. Since the cardioprotective nature of GLP-1 mimetics is typically examined after long term exposure, it is also possible that GLP-1 has multiple tissue specific intracellular signaling cascades based on time of exposure or concentration (Jessen et al., 2017; Tomas et al., 2020).

## Conclusion

Given the long history of both basic science and clinical investigations into cardiac autonomic function, it can be easy to assume that we fully understand how these systems work. While we know a great deal about the anatomy of these circuits, how they process the wide variety of complex signals they receive and ultimately integrate and relay this information to peripheral organs, such as the heart, is still under active investigation. Despite the scarcity of studies investigating the effect of metabolic signaling on cardiac vagal motor neuron physiology, reports have confirmed the therapeutic potential—although varied in magnitude—of activating vagal pathways, most notably through vagal stimulation. Although these results may provide a mechanistic rationale for the importance of cardiac-related vagal tone and health, more studies investigating the plasticity within autonomic regulatory circuits related to various metabolic cues are needed to better understand the fundamental role of vagal signaling in metabolic and cardiovascular physiology.

During this discussion, it is also important to consider that the plasticity associated with a disease may not simply be an exaggeration of normal physiology. Therefore, considerable work must be done to determine the role of the brain in mediating cardiometabolic integrative homeostasis in both health and disease. These examinations into autonomic contributions to cardiometabolic function need to include time courses throughout disease progression. This will determine when important neuronal remodeling occurs, revealing important biological milestones for intervention. Continued investigation into these autonomic pathways will not only increase our understanding of these circuits, but will develop a more informed perspective that will influence current clinical treatment guidelines for patients to provide early and reliable detection markers of autonomic dysregulation, as well as a more complete management of a patient’s disease and the prevention of cardiac-related morbidity and mortality.

## Author Contributions

LE, SF, and CB wrote and edited the manuscript. All authors contributed to the article and approved the submitted version.

## Conflict of Interest

The authors declare that the research was conducted in the absence of any commercial or financial relationships that could be construed as a potential conflict of interest.
